# Antiepileptic Drug Use, Falls, Fractures, and BMD in Postmenopausal Women: Findings From the Women's Health Initiative (WHI)

**DOI:** 10.1359/jbmr.091027

**Published:** 2009-10-17

**Authors:** Laura D Carbone, Karen C Johnson, John Robbins, Joseph C Larson, J David Curb, Kathleen Watson, Margery Gass, Andrea Z LaCroix

**Affiliations:** 1 Department of Veterans Affairs Medical CenterMemphis, TN, USA; 2 Department of Medicine, University of Tennessee Health Science CenterMemphis, TN, USA; 3 Department of Preventive Medicine, University of Tennessee Health Science CenterMemphis, TN, USA; 4 Department of Medicine, University of CaliforniaDavis, CA, USA; 5 Fred Hutchinson Cancer Research CenterSeattle, WA, USA; 6 Department of Geriatric Medicine, John A Burns School of Medicine, University of HawaiiHonolulu, HI, USA; 7 University of WashingtonSeattle, WA, USA; 8 University of CincinnatiCincinnati, OH, USA

**Keywords:** population studies, aging, menopause

## Abstract

Antiepileptic drugs (AEDs) are used increasingly in clinical practice to treat a number of conditions. However, the relationship between the use of these medications, particularly the newer AEDs, and fracture risk has not been well characterized. We used data from the Women's Health Initiative (WHI) to determine the relationship bewteen the use of AEDs and falls, fractures, and bone mineral density (BMD) over an average of 7.7 years of follow-up. We included 138,667 women (1,385 users of AEDs and 137,282 nonusers) aged 50 to 79 years in this longitudinal cohort analyses. After adjustment for covariates, use of AEDs was positively associated with total fractures [hazard ratio (HR) = 1.44, 95% confidence interval (CI) 1.30–1.61], all site-specific fractures including the hip (HR = 1.51, 95% CI 1.05–2.17), clinical vertebral fractures (HR = 1.60, 95% CI 1.20–2.12), lower arm or wrist fractures (HR = 1.40, 95% CI 1.11–1.76), and other clinical fractures (HR = 1.46, 95% CI 1.29–1.65) and two or more falls (HR = 1.62, 95% CI 1.50–1.74) but not with baseline BMD or changes in BMD (*p* ≥ .064 for all sites). Use of more than one and use of enzyme-inducing AEDs were significantly associated with total fractures (HR = 1.55, 95% CI 1.15–2.09 and HR = 1.36, 95% CI 1.09–1.69, respectively). We conclude that in clinical practice, postmenopausal women who use AEDs should be considered at increased risk for fracture, and attention to fall prevention may be particularly important in these women. © 2010 American Society for Bone and Mineral Research.

## Introduction

Worldwide, 50 million people suffer from epilepsy, and the majority use antiepileptic drugs (AEDs).(1) However, increasingly, even among those without epilepsy, use of AEDs is becoming more prevalent.(2,3) Therefore, the relationship of AED use to osteoporosis is of interest.

The relationship of AED use to fractures is controversial, with some,(4,5) although not all,(6) studies suggesting that fracture risk is increased. However, these reports are limited by sample size, selection of controls, and most important, the failure to take into account potential confounding factors.(4,5) Even a large pharmacoepidemiologic database that examined the relationship of AED use and fractures was unable to control for important potential confounders, including family history of fractures, smoking history, and calcium/vitamin D intake,(7) which are available in the Women's Health Initiative (WHI). In addition, most studies of the relationship of AED use and fractures have excluded racial groups other than Caucasians.(6,8) However, epilepsy does not have a particular racial predilection.(9) Few studies have included the non-enzyme-inducing category of these drugs,(7,10,11) whose use is increasing rapidly, including for indications other than epilepsy.(2,3) Finally, fall history has not been well considered in previous studies of the relationship between AED use and fracture

There are a number of mechanisms by which AEDs may be associated with fractures. Vitamin D deficiency may occur with AEDs,(12–18) and it has been suggested that enzyme-inducing AEDs (inducers of the cytochrome P450 enzyme system) in particular may be associated with vitamin D deficiency because enzyme induction may lead to increased catabolism of vitamin D.(19) In addition, undercarboxylation of the bone Gla protein osteocalcin, a vitamin K–dependent protein that is a specific product of the osteoblasts,(20) has been reported with AEDs.(4) This is potentially important because epidemiologic studies suggest an association between undercarboxylation of osteocalcin and increased fracture rates.(21,22) Fall history is important because falls may be associated with AEDs and/or the condition for which the AED is prescribed. Dizziness, ataxia, and unsteady gait are among the most common adverse effects of AEDs,(23–27) thereby having an impact on fall risk. Alternatively, conditions such as seizures(1) and diabetic neuropathy(28) are treated with AEDs, and falls occur as a result of these conditions.(29)

No study to date has examined the relationship between AED use and bone mineral density (BMD) and fractures (including fall history) within the context of a large prospective study of ambulatory, multiethnic, noninstitutionalized women that uniformly collected medication exposure, BMD, and fracture and fall outcomes. The purpose of this study was to examine the association between AED drug use and changes in BMD, fractures, and falls in the WHI, including use of more than one AED and use of the more recently introduced non-enzyme-inducing AEDs.

We hypothesized that compared with nonusers, AED users would have greater loss in BMD, more falls, and more fractures. Additionally, we hypothesized that users of more than one AED as opposed to users of one AED would have more fractures and that there would be more fractures in users of enzyme-inducing AEDs compared with non-enzyme-inducing AEDs. We suspected that this would be the case because increased hepatic catabolism of vitamin D metabolites occurs with enzyme-inducing AEDs.(30)

## Materials and Methods

### Design/setting and participants

We conducted a prospective study of AED use and changes in BMD (3 years) and fractures and falls (7.7 years) in women aged 50 to 79 years at 40 clinical centers in the WHI from 1993 to 1998. Users of corticosteroids (*n* = 1406) and participants with missing covariate data (*n* = 21,735) were excluded. The study population for the fracture outcomes (1385 AED users and 137,282 nonusers of AEDs) included all women in the WHI Observational Study (OS) and the WHI Clinical Trials (CT). BMD was available in a subset (84 AED users and 8677 nonusers of AEDs). Details of WHI methods have been described elsewhere.(31) All protocols were reviewed and approved by the institutional review board at each participating center.

### Medication use

Current medication use was ascertained by having the participants bring the containers with all medications taken for the prior 2 weeks to the baseline visit and the year 3 visit. Interviewers entered each medication into the database, which assigned drug codes using Medispan software (First DataBank, Inc., San Bruno, CA, USA). Information on duration of use but not dose was recorded. For this study, we defined hormone therapy (HT) as use of an estrogen with or without progesterone (oral or patch formulations). Use of bisphosphonates, calcitonin, and selective estrogen receptor modulators (SERMS) also was noted. Current AED use was defined as any use at the baseline and/or at the year 3 visit. We categorized AEDs into two groups: (1) enzyme-inducing and (2) non-enzyme-inducing. The category of enzyme-inducing AEDs included carbamazepine, mephenytoin, phenytoin, and primidone, and the category of non-enzyme-inducing AEDs included clonazepam, divalproex sodium, valproic acid, gabapentin, lamotrigine, methsuxime, and topiramate.(32–34) Since divalproex sodium and valproic acid may be weakly cytochrome P450 enzyme-inducing and topiramate may be weakly enzyme-inducing in a dose-dependent manner,(35) these medications also were included as enzyme inducers in a separate sensitivity analysis. In models looking at the number of AEDs used, AED users were split into two groups, those who used only one AED and those who used more than one AED. These levels of AED use were put into a model along with those who were not using AEDs. Duration of use of AEDs was examined in three categories (<2 years, 2 to 5 years, and >5 years).

### Dietary supplements

Dietary intake of calcium and vitamin D were measured with a semiquantitative food-frequency questionnaire.(36) Vitamin D intake was defined as the sum of vitamin D from supplements and diet, whereas calcium intake included all calcium from medications, supplements, and diet.

### Other covariates

Questionnaires were used to collect information on age, ethnicity, smoking history, parental history of hip fractures, prevalent fractures on or after age 55, age at menopause, history of Parkinson's disease, history of diabetes, history of multiple sclerosis (MS), history of stroke, and health status. A history of falls was defined as two or more falls in the year before the baseline visit. Alcohol consumption was estimated from the food-frequency questionnaire. Energy expenditure from recreational physical activity was used to determine physical activity levels. The 36 Item Short Form Health Survey (SF-36) was used to calculate a physical function construct (scale 0 to 100), with higher scores indicating better physical function. Height and weight were measured in the WHI(31) and used to calculate body mass index (BMI). Geographic study sites and trial enrollment [clinical trials (CTs) or observational study (OS)] were adjusted for in the statistical analyses. Within the calcium/vitamin D supplementation trial, adjustments were made for assignments to placebo versus calcium/vitamin D supplementation. All covariates were selected pre hoc; of these, age, race, and ethnicity were included in minimally adjusted models, and all covariates were included in fully adjusted models. All covariates used were from the baseline visit.

### Outcomes and follow-up

#### Measurement of BMD

BMDs of the total hip, anteroposterior lumbar spine, and total body were measured at baseline and year 3 in participants at 3 of the 40 clinical centers of the WHI (Pittsburgh, Pennsylvania, Birmingham, Alabama, and Phoenix/Tucson, Arizona) by dual-energy X-ray absorptiometry (DXA) using a Hologic QDR densitometer (Model 2000, 2000+, or 4500 Fan-beam, Hologic, Inc., Waltham, MA, USA) Three Hologic phantoms (spine, hip, and linearity) were exchanged among these three centers and measured in array mode five times, once each day for five consecutive days, to assess cross-calibration. Spine, hip, and linearity phantoms were in close agreement (interscanner variability < 1.5% for the spine, 4.8% for the hip, and 1.7% for linearity). We determined change in BMD by AED use category at these sites from baseline to year 3.

#### Fracture ascertainment

In WHI CTs, all fracture outcomes were verified by radiology reports. Radiographs were not obtained to ascertain subclinical vertebral fractures.(37) In the WHI OS, only hip fractures, and not any other fractures, were adjudicated by central review of radiology reports.

#### Fall ascertainment

Incident falls were ascertained prospectively using questionnaires asking about the number of times the participant fell (excluding falls owing to sports) in the interval since the last medical history update.

### Statistical analysis

Descriptive analyses (Tables 1 and 2) were stratified by AED use at baseline and are presented with the number of participants, means, and standard deviations in each group for continuous variables and frequencies with percentages for categorical covariates. For continuous covariates, a two sample *t* test was used to compare between groups, whereas for categorical variables, a chi-square test was used to assess significance. Modeling (Tables 3 and 4) was done using Cox proportional hazards models and includes results both with minimal adjustment (e.g., age, race/ethnicity, and BMI) and full adjustment (i.e., age, race/ethnicity, BMI, smoking status, alcohol intake, calcium and vitamin D intake, prevalent fracture at age 55+, history of falls, bisphosphonate use, past/current use of HT, SERM use, calcitonin use, age of menopause, physical activity levels, physical function construct, history of diabetes, history of stroke, parental history of hip fractures, study site region, self-reported health, history of MS, and history of Parkinson's disease). All proportional hazards models additionally were adjusted for intervention status in the WHI hormone therapy (active/placebo/not randomized), dietary modification (intervention/comparison/not randomized), and calcium/vitamin D (active/placebo/not randomized) trials, as well as enrollment in the WHI OS (yes/no).

**Table 1:** Characteristics of Study Population (Demographics)

	Antiepileptic users		Nonusers		
			
	*n*	Mean (SD) or %	*N*	Mean (SD) or %	*p* Value
Age at screening, mean (SD)	1385	63.06 (7.34)	137,282	63.22 (7.19)	.427
50–59	478	34.5	45,367	33.0	
60–69	601	43.4	62,045	45.2	
70–79	306	22.1	29,870	21.8	
Race/ethnicity					.002
White	1212	87.5	114,725	83.6	
Black	86	6.2	11,509	8.4	
Hispanic	46	3.3	4,889	3.6	
Native American	5	0.4	549	0.4	
Asian/Pacific Islander	20	1.4	3,769	2.7	
Unknown	16	1.2	1,841	1.3	
Smoking					.002
Never smoked	682	49.2	69,654	50.7	
Past smoker	576	41.6	58,279	42.5	
Current smoker	127	9.2	9,349	6.8	
Alcohol use					<.001
Nondrinker	614	44.3	39,542	28.8	
Current drinker, <7 drinks/week	662	47.8	81,296	59.2	
Current drinker, 7+ drinks/week	109	7.9	16,444	12.0	
HT use					<.001
Never used	466	33.6	56,639	41.3	
Past user	274	19.8	22,680	16.5	
Current user	645	46.6	57,963	42.2	
Bisphosponate use	43	3.1	2,748	2.0	.004
Calcitonin use	9	0.6	383	0.3	.010
History of falls	352	25.4	16,429	12.0	<.001
History of fracture on/after age 55	242	17.5	17,888	13.0	<.001
Parent with hip fracture after age 40	10	0.7	747	0.5	.371
Self-reported health status					<.001
Excellent	110	7.9	23,988	17.5	
Very good	432	31.2	57,064	41.6	
Good	529	38.2	44,693	32.6	
Fair	267	19.3	10,637	7.7	
Poor	47	3.4	900	0.7	
History of treated diabetes	78	5.6	5,810	4.2	.010
History of stroke	79	5.7	1,687	1.2	<.001
History of multiple sclerosis (MS)	19	1.4	375	0.3	<.001
History of Parkinson's disease	13	0.9	234	0.2	<.001
CT versus OS^a^					<.001
CT randomized	488	35.2	55,158	40.4	
OS Enrolled	897	64.8	82,124	59.8	
HT trial group					.093
HT not randomized	1195	86.3	115,562	84.2	
HT placebo	98	7.1	10,824	7.9	
HT active	92	6.6	10,896	7.9	
Dietary modification trial group					.004
DM not randomized	1042	75.2	97,670	71.1	
DM control	206	14.9	23,812	17.3	
DM intervention	137	9.9	15,800	11.5	
Calcium/vitamin D trial group					.005
CaD not randomized	1136	82.0	107,698	78.5	
CaD intervention	122	8.8	14,811	10.8	
Total MET-hours/week of recreational activity, mean (SD)	1385	11.25 (13.44)	137,282	12.57 (13.74)	<.001
<2.3	409	29.5	34,093	24.8	
2.3 to <8.4	341	24.6	34,407	25.1	
8.4 to <18.5	348	25.1	35,194	25.6	
≥18.5	287	20.7	33,588	24.5	
Baseline total vitamin D intake, mean IU/day (SD)	1385	393.0 (287.2)	137,282	374.7 (280.1)	.015
<200 IU	488	35.2	51,035	37.2	
200 to <400 IU	232	16.8	24,847	18.1	
400 to <600 IU	370	26.7	34,503	25.1	
≥600 IU	295	21.3	26,897	19.6	
Baseline total calcium intake, mean (SD)	1385	1227.6 (761.1)	137,282	1191.2 (748.1)	.071
<800 mg	451	32.6	46,300	33.7	
800 to <1200 mg	331	23.9	33,609	24.5	
≥1200 mg	603	43.5	57,373	41.8	
Height, cm, mean (SD)	1385	162.16 (6.25)	137,282	161.87 (6.48)	.094
Weight, kg, mean (SD)	1385	73.58 (16.61)	137,282	73.04 (16.09)	.221
Body-mass index (BMI), kg/m^2^, mean (SD)	1385	27.99 (6.22)	137,282	27.87 (5.91)	.443
Age at menopause, years, mean (SD)	1385	46.56 (7.09)	137,282	48.11 (6.40)	<.001

**Table 2:** Baseline Medication Use in Study Population by Antiepileptic Drug Use

	Anticonvulsant users (*n* = 1385)		Nonusers (*n* = 137,282)		
			
	*n*	%	*N*	%	*p* Value
HT use at baseline					<.001
Never used	466	33.6	56,639	41.3	
Past user	274	19.8	22,680	16.5	
Current user	645	46.6	57,963	42.2	
Bisphosphonate use	43	3.1	2,748	2.0	.004
Calcitonin use	9	0.6%	386	0.3%	.010
Selective estrogen receptor modulator (SERM) use	1	0.1	38	0.0	.326

**Table 3:** Fracture Incidence and Fall Frequency by Use of AEDs at Baseline

	Number of Events	Annual %	Model 1^a^ HR^c^ (95% CI)	Model 2^b^ HR^c^ (95% CI)
Hip fractures				
Nonuser	1,532	0.15	1.00	1.00
User	30	0.29	1.98 (1.38–2.85)	1.51 (1.05–2.17)
<2 years	10	0.30	2.19 (1.18–4.08)	1.59 (0.85–2.97)
2 to 5 years	9	0.27	1.72 (0.89–3.32)	1.31 (0.68–2.53)
>5 years	11	0.31	2.07 (1.14–3.74)	1.63 (0.90–2.95)
Clinical vertebral fractures				
Nonuser	2,355	0.22	1.00	1.00
User	49	0.48	2.15 (1.62–2.86)	1.60 (1.20–2.12)
<2 years	17	0.51	2.38 (1.48–3.84)	1.69 (1.05–2.73)
2 to 5 years	12	0.37	1.61 (0.91–2.85)	1.17 (0.66–2.07)
>5 years	20	0.56	2.45 (1.58–3.80)	1.92 (1.23–2.98)
Lower arm or wrist				
Nonuser	5,034	0.48	1.00	1.00
User	74	0.72	1.52 (1.21–1.91)	1.40 (1.11–1.76)
<2 years	15	0.45	0.95 (0.57–1.58)	0.90 (0.54–1.49)
2 to 5 years	28	0.83	1.79 (1.23–2.60)	1.64 (1.13–2.38)
>5 years	31	0.87	1.79 (1.26–2.55)	1.63 (1.14–2.32)
Other clinical fractures				
Nonuser	15,974	1.51	1.00	1.00
User	256	2.49	1.73 (1.53–1.96)	1.46 (1.29–1.65)
<2 years	86	2.56	1.76 (1.42–2.17)	1.48 (1.19–1.83)
2 to 5 years	71	2.11	1.44 (1.14–1.82)	1.21 (0.96–1.53)
>5 years	99	2.79	1.99 (1.63–2.42)	1.69 (1.38–2.06)
Total fractures				
Nonuser	22,137	2.10	1.00	1.00
User	344	3.35	1.70 (1.53–1.89)	1.44 (1.30–1.61)
<2 years	107	3.18	1.60 (1.32–1.93)	1.36 (1.12–1.64)
2 to 5 years	103	3.06	1.53 (1.26–1.85)	1.29 (1.07–1.57)
>5 years	134	3.78	1.96 (1.66–2.33)	1.68 (1.42–1.99)
Two or more falls				
Nonuser	45,246	4.29	1.00	1.00
User	760	7.40	2.15 (2.00–2.31)	1.62 (1.50–1.74)
<2 years	257	7.65	2.23 (1.97–2.52)	1.58 (1.40–1.79)
2 to 5 years	239	7.10	2.05 (1.80–2.33)	1.55 (1.36–1.76)
>5 years	264	7.44	2.16 (1.92–2.44)	1.71 (1.52–1.94)

a Model 1 adjusted for age, ethnicity, BMI, and WHI trial participation and intervention.

b Model 2 adjusted for age, race/ethnicity, BMI, smoking, alcohol, calcium and vitamin D intake, history of fractures (fracture at age 55+), history of falls (two or more in the year prior to enrollment), bisphosphonates, past/current use of HT, SERMs, calcitonin, age of menopause, physical activity levels, physical function construct, diabetes, stroke, parental history of hip fractures, study site region, self-reported health, multiple sclerosis, Parkinson's, WHI trial participation and intervention.

^bc^ Nonusers of AEDs are the reference group for both the any-use and the duration models.

**Table 4:** Relationship of AED Use With Total Fractures by Number and Type of AED Used

	Events	Annual %	Model 1 HR (95% CI)^a^	Model 2 HR (95% CI)^b^
Nonuser	22,137	2.10	1.00	1.00
Single AED user	293	3.18	1.60 (1.42–1.79)	1.37 (1.22–1.54)
Multiple AED user	51	4.75	2.68 (2.04–3.53)	2.12 (1.61–2.80)
Non-enzyme AED user	138	2.86	1.44 (1.22–1.71)	1.20 (1.02–1.42)
Enzyme AED user	190	3.69	1.87 (1.62–2.15)	1.64 (1.42–1.89)
Both AED type user	16	5.39	3.02 (1.85–4.93)	2.10 (1.28–3.43)

a Model 1 adjusted for age, ethnicity, BMI, and WHI trial participation and intervention.

b Model 2 adjusted for age, race/ethnicity, BMI, smoking, alcohol, calcium and vitamin D intake, prevalent fractures (fracture at age 55+), prevalent falls (two or more in the year prior to enrollment), bisphosphonates, past/current use of HT, SERMs, calcitonin, age of menopause, physical activity levels, physical function construct, diabetes, stroke, parental history of hip fractures, study site region, self-reported health, MS, Parkinson's, and WHI trial participation and intervention.

Results for each outcome are presented with event totals, annualized percentages, and hazard ratios (HRs) with their corresponding 95% confidence intervals (CIs). Fracture outcomes were modeled individually first by any AED use at baseline (users versus nonusers) and then by duration of AED use at baseline (see Table 3; stratified into groups of <2years, 2 to 5 years, >5 years versus nonusers).

Subgroup analyses also were performed, duplicating the main models while separating out users by number and type (see Table 4) of AED used. A final model was run comparing enzyme-inducing AED users with non-enzyme-inducing AED users, excluding the 41 persons who used both enzyme-inducing and non-enzyme-inducing AEDs.

BMD means by AED use are presented with sample sizes, means, and standard errors in Table 5. Comparisons between the means at the initial measurement (baseline), final measurement (year 3), and the 3-year change in BMD between the AED user and nonusers groups were done both in minimally and fully adjusted linear models, modeling the BMD covariate of interest by baseline AED use and using the baseline covariates listed earlier for adjustments. All analyses were conducted with SAS Version 9.1 (SAS Institute Inc., Cary, NC).

**Table 5:** Mean Bone Mineral Density (BMD, g/cm^2^ ± SE) and % Changes ± SE in Mean BMD by Current AED Use

	Antiepileptic user		Nonuser			
				
	*n*	Mean^a^ (SE)	*n*	Mean* (SE)	*p* Value^a^	*p* Value^b^ (adjusted)
Initial measurement						
Total hip	83	0.84 (0.017)	8644	0.87 (0.007)	.096	.224
Total spine	82	0.96 (0.020)	8392	0.98 (0.008)	.276	.647
Total body	83	1.01 (0.013)	8645	1.02 (0.005)	.304	.777
Final measurement						
Total hip	60	0.86 (0.020)	7222	0.89 (0.008)	.127	.289
Total spine (final)	60	0.98 (0.025)	7017	1.01 (0.010)	.097	.308
Total body (final)	60	1.01 (0.016)	7190	1.04 (0.006)	.095	.229
3-Year BMD change						
% Change in total hip	58	0.52 (0.576)	7141	1.24 (0.220)	.180	.312
% Change in total spine	58	4.15 (0.730)	6962	2.91 (0.283)	.067	.064
% Change in total body	57	1.34 (0.535)	7119	1.42 (0.203)	.870	.539

a Adjusted for WHI trial participation and intervention.

b Adjusted for age, race, BMI, smoking, alcohol, calcium and vitamin D intake, history of fractures (fracture at age 55+), history of falls (two or more in year prior to enrollment), bisphosphonates, past/current use of HT, SERMs, calcitonin, age of menopause, physical activity levels, physical function construct, diabetes, stroke, parental history of hip fractures, study site region, self-reported health, multiple sclerosis, Parkinson's, WHI trial participation, and intervention.

## Results

### Baseline characteristics and medication use

Our data set included 1385 AED users (including *n* = 269 carbamazepine; *n* = 470 clonazepam; *n* = 110 divalproex sodium; *n* = 81 gabapentin; *n* = 5 lamotrigine; *n* = 3 mephenytoin; *n* = 3 methsuximide; *n* = 370 phenytoin; *n* = 67 primidone; *n* = 1 topiramate; and *n* = 6 valproic acid). Baseline characteristics of the study population and medication use by AED use are shown in Tables 1 and 2, respectively. In general, AED users were sicker and had more risk factors for fracture than nonusers (see Table 1). However, AED users were more likely to be HT, bisphosphonate, or calcitonon users than were nonusers (see Table 2). Among AED users, there was no significant association of duration of use of AEDs with prevalent fractures (data not shown).

### Fracture and fall outcomes in whole cohort

Among AED users, the annualized percentage of incident fractures was 3.35% for total fractures, 0.29% for hip fractures, 0.48% for clinical vertebral fractures, 0.72% for lower arm/wrist fractures, 2.49% for other clinical fractures, and 7.40% for two or more falls. In nonusers of AEDs, the respective annualized percentages for these incident fractures and falls were 2.10%, 0.15%, 0.22%, 0.48%, 1.51%, and 4.29%, respectively (see Table 3). In models adjusted for age, race/ethnicity, and BMI and in fully adjusted models, respectively, use of AEDs was positively associated with total fractures, all site-specific fractures, and falls (see Table 3). In fully adjusted models, there was a significant association of duration of use of AEDs and total fractures, two or more falls, and all site-specific fractures (*p* ≤ .005 for all), except for hip fractures (*p* = .156) (data not shown). There was no significant interaction between race/ethnicity and total fractures (*p* = .75) (data not shown).

### Fracture and fall outcomes by number of AEDs used

In models adjusted for age, race/ethnicity, and BMI compared with nonusers of AEDs, users of a single AED and more than one AED were both at significantly higher risk for total fractures (HR = 1.60, 95% CI 1.42–1.79 and HR = 2.68, 95% CI 2.04–3.53), respectively; this persisted in fully adjusted models (HR = 2.68, 95% CI 2.04–3.53 and HR = 2.12, 95% CI 1.61–2.80, respectively; see Table 4). In models adjusted for age, race/ethnicity, and BMI and in fully adjusted models respectively, HRs comparing the 150 participants who reported use of two or more AEDS with users of a single AED (*n* = 1235) were significantly elevated for total fractures (HR = 1.68, 95% CI 1.25–2.26 and HR = 1.55, 95% CI 1.15–2.09).

### Fracture and fall outcomes by use of enzyme-inducing and non-enzyme-inducing AEDs

In models adjusted for age, race/ethnicity, and BMI compared with nonusers of AEDs, users of enzyme-inducing AEDs (*n* = 681), non-enzyme-inducing AEDs (*n* = 663), and both enzyme-inducing and non-enzyme-inducing AEDs (*n* = 41) were at significantly higher risk for total fractures (HR = 1.44, 95% CI 1.22–1.71; HR = 1.87, 95% CI 1.62–2.15; and HR = 3.02, 95% CI 1.85–4.93, respectively); this remained true in fully adjusted models (HR = 1.20, 95% CI 1.02–1.42; HR = 1.64, 95% CI 1.42–1.89; and HR = 2.10, 95% CI 1.28–3.43, respectively; see Table 4). Compared with non-enzyme-inducing AED users (*n* = 663), in models adjusted for age, race/ethnicity, and BMI, users of enzyme-inducing AEDs (*n* = 681) were at significantly higher risk of total fractures (HR = 1.29, 95% CI 1.04–1.61). In fully adjusted models, a similar increased risk of total fractures also was seen (HR = 1.36, 95% CI 1.09–1.69, respectively). These results were not materially changed when divalproex sodium, valproic acid, and topiramate were included as enzyme-inducing rather than as non-enzyme-inducing AEDs (data not shown).

### BMD outcomes

In the subset of women with BMD, in fully adjusted models, compared with nonusers of AEDs, users of AEDs had no significant differences in percentage change from baseline to year 3 BMD of the hip (*p* = .312), spine (*p* = .064), or total body (*p* = 0.539; see Table 5).

## Discussion

In this largest prospective study of AED use and osteoporosis in multiethnic postmenopausal women, AEDs were associated with a significantly increased risk for total and site-specific fractures, including both nonvertebral (including hip) and clinical vertebral fractures. The risk for fractures was a function of the number and type of AEDs used, with women who used more than one AED as opposed to single AED use and those who used enzyme-inducing AEDs as opposed to the more recently introduced non-enzyme-inducing AEDs more likely to fracture. There was no significant association of AED use with BMD changes; there was, however, a significant association of AED use with falls.

The more than 1300 postmenopausal women AED users in the WHI is the largest cohort of ambulatory, noninstitutionalized, multiethnic women ever prospectively examined for fractures. Although an elevated risk for total fractures has been reported previously in AED users, these studies were limited by the inability to control for potential confounders(5,7,11) that we were able to consider in the WHI.

Over 11% of our population (>200 AED users) were of racial groups other than white (predominantly black), and the increased risk of total fractures in these women was similar to that of white women. This is potentially important because seizures, a major indication for use of AEDs, occur across all racial groups,(9) and the relationship between AED use and fractures in multiracial groups has not, to our knowledge, been explored previously. Others have suggested that black race is an independent risk factor for receipt of use of the older, enzyme-inducing AEDs.(38)

Relative to site-specific fractures, in agreement with our study, some,(7,8) but not all,(6) studies report an elevated risk for hip fractures with AED use, although these studies, in contrast with the WHI, did not include racial groups other than Caucasians.(6–8) In accord with our results, an increased risk for Colles' fracture with AED use has been documented.(7,39) The relationship between AED use and vertebral fractures is more controversial, with one study, similar to ours, reporting an approximately 50% increased relative risk for vertebral fractures with AED use,(7) whereas another study found no significant relationship between AED use and vertebral fractures.(39) Our study differs from those,(7,39) however, in that we included only clinical vertebral fractures.

In the WHI, both the number (more than one compared with one) and type (enzyme-inducing compared with non-enzyme-inducing) of AED used were significantly associated with fractures. In agreement with this, one study(7) found that relative to non-enzyme-inducing AEDs, enzyme-inducing AEDs were more likely to be associated with fractures. However, others have noted no difference in the relationship between the type of AED used and fracture risk.(38) Although we were not able to explore the mechanisms by which enzyme-inducing AEDs were associated with more fractures than non-enzyme-inducing AEDs in our study, we suspect that this may have been driven by differences in 25-hydroxyvitamin D levels [25(OH)D], which we did not have measurements of. Differences in 25(OH)D levels between enzyme-inducing AEDs and non-enzyme-inducing AEDs may have been particularly important because this could have had an impact on both BMD(40) and recurrent falls.(41)

Cross sectionally, in the WHI there were no significant differences in BMD between the 113 women who used AEDs and the nonusers of AEDs. In contrast, other cross-sectional studies have reported lower BMDs of the lumbar spine and hip with AED use.(40–42) Similarly, in the WHI, differences in fracture rates between AED users and nonusers were not explained by longitudinal differences in changes in BMD. However, significant rates of bone loss at the hip in both women(22) and men(43) AED users have been reported by others. The reasons for these differences in findings in BMD in users of AEDs in the WHI compared with these other studies are not clear. It is possible that it is because only a subset of AED users (84 women) over a limited time (3 years) had BMD measured, limiting our power to detect BMD differences between the groups. This BMD population was slightly older and had a larger percentage of minority women than the whole cohort. Another important mechanism that may underlie the relationship of AED use and fractures is changes in bone metabolism; however, biomarkers of bone metabolism were not available for this entire cohort. Alternatively, it is possible that AEDs increase the risk of fracture solely by increasing falls and have no effects on either BMD or biomarkers. If so, this may operate in several ways. The first mechanism would be a direct result of the AED itself increasing falls because dizziness, ataxia, and unsteady gait are among the most common side effects of AEDs.(23–26) The second potential mechanism would not be as a direct effect of the AED by rather confounding by indication because an increasing number of falls may occur in medical conditions for which AEDs are used.(1)

There are a number of limitations to our study. Although we adjusted for conditions including diabetes, multiple sclerosis, and Parkinson's disease in which AEDs may be used, we did not have information on seizure history, and seizures may be associated with fractures.(39) In addition, doses of AEDs were not captured in the WHI. Prefracture health, which may change precipitously in older populations, is a predictor of fracture,(42,43) and we only included baseline levels of prefracture health as a covariate in the models. 25(OH)D may be associated with BMD(44) and AED use,(15) but we could not adjust for this. The limited number of women who had DXAs may have contributed to our inability to show an association between AEDs and BMD.

In conclusion, there was a significant association between AED use and falls and fractures in multiracial postmenopausal women in the WHI. The risk for total fractures and all site-specific fractures including nonvertebral (and specifically hip) and clinical vertebral fractures was significantly higher in AED users. Enzyme-inducing AEDs were associated with the highest fracture risk. Whether the association of AED with fractures is a function of the drug itself or rather the condition for which it is being prescribed merits further study. In clinical practice, however, postmenopausal women who use AEDs should be considered at risk for fracture, and attention to fall prevention in these women may be particularly important.
